# Lead Poisoning and the Dangers of Pragmatism

**DOI:** 10.3390/ijerph15091997

**Published:** 2018-09-13

**Authors:** Daniel Renfrew

**Affiliations:** Department of Sociology and Anthropology, West Virginia University, Morgantown, WV 26506-6326, USA; daniel.renfrew@mail.wvu.edu

**Keywords:** lead poisoning, LMICs, Uruguay

## Abstract

Drawing from ethnographic research on lead poisoning in Uruguay and secondary literature from lead poisoning cases around the world, the commentary argues that public health policy guided by pragmatism presents multiple dangers to effective health intervention.

In the past few years, lead poisoning has once again drawn extensive news coverage and sparked widespread public concern in the United States. The Flint, Michigan water crisis serves as a reminder of lead’s stubborn persistence in urban infrastructure and the toxic legacies of industrial abandonment [1]. It also reveals how environmental health crises are profoundly social and political in character. Lead contamination in Flint was a product of the misguided, tragic and even criminal decisions made by local and state political, environmental, and public health officials. Its contours were shaped by a political climate of fiscal austerity and the structurally disproportionate exposure to harm experienced by urban minorities and the poor [2]. In the aftermath of Flint, a Reuters investigative report revealed over 3000 U.S. community “hot spots” with even graver cases of lead poisoning than those found during the peak of Flint’s water crisis [3]. The pervasive hazards of lead in water and the interior lead paints in old housing stock are the two primary sources of continued lead contamination across the country, and both disproportionately affect the urban poor. A recent Consumer Reports study, however, revealed high levels of lead in popular baby food brands, even in the organic labels catering to middle and upper-middle class parents, reminding us once again that lead exposure has never been the exclusive domain of the poor, but has always posed a universal risk as well [4]. Public, political and even scientific recognition of this silent scourge, however, has typically been conditioned by the interplay of broader socio-economic contexts and political forces. Lead poisoning, quite simply, has cyclically appeared and faded from public view over the past two thousand years for reasons having little to do with its objective presence in the environment or in people’s bodies [5].

How are the social and political experiences of lead contamination and poisoning similar or different in low and middle-income countries (LMICs)? I would argue that there are key differences that must be recognized, including typically higher levels of acute lead poisoning, later phaseout of tetraethyl leaded (TEL) gasoline, and greater relative contributions to contamination from the informal economy. But I would also caution that these differences sometimes obscure the similarities of experience and more importantly the interconnectedness and shared complicity of high income and LMICs, or what many social scientists refer to, respectively, as the global North and the global South. These include the globally interconnected toxic cycle of mining, production, consumption, waste disposal and recycling. My own long-term ethnographic research on lead poisoning in Montevideo, Uruguay has sought to unveil the interplay of social, political, economic and scientific forces that led to the creation and public recognition of a mass lead poisoning epidemic in 2001, as well as the new forms of activism, knowledge production, and social and environmental regulation and governance that emerged in response to the lead discoveries [6,7].

Lead contamination was discovered in Uruguay against the backdrop of a deep economic crisis and prolonged and unpopular neoliberal reforms that led to deindustrialization, deregulation and downsizing. During this period, unemployment reached record numbers of over 20%, unprecedented levels of extreme poverty and indigence devastated urban and rural communities, and the traditional political parties that had ruled the country for all of its modern history had become widely delegitimized and bankrupt, paving the way for the historic rise to power of the center-left Broad Front coalition [7,8]. Lead contamination had been a material reality for decades, but the crisis put more people in harm’s way, particularly through the precipitous growth of urban squatter settlements, some of them located on lots and riverbanks long used as industrial dumping grounds, and the growth of the informal economy, some of it dedicated to contaminating e-waste and garbage recycling. Other universal sources of exposure included the persistent use of tetraethyl leaded gasoline (until its phaseout in 2004), paints, and water pipes and fixtures. Lead was first discovered in the proud and militant western Montevideo industrial neighborhood of La Teja, where a novel environmental justice-style movement was able to tap into the neighborhood’s established networks of activism and community solidarity, while forging strategic connections with the mass media to make the story “explode” across the Uruguayan news cycle for months and even years. Lead poisoning struck a compelling chord, I have argued, as a symbolic stand-in for the broader social, economic and political crises afflicting the nation [7].

With a growing social movement and persistent media pressure demanding state action and industrial accountability, in 2001 the national government formed an inter-institutional commission made up of representatives of various ministries, municipalities, state enterprises, academic programs and civil society. The Public Health Ministry opened the nation’s first specialized lead poisoning clinic and directed remediation and harm reduction measures, while the Housing Ministry coordinated mass relocations from contaminated squatter settlements to new government-built housing developments, and Parliament introduced important lead control legislation. These efforts, funded by and coordinated through the state’s Public Health Ministry, reflected an unprecedented and ambitious public health initiative that coordinated all three branches of state power and advocated for universal lead screening, the elimination of lead sources, mass housing relocation and aggressive remediation.

By around 2003 or 2004, however, the state had both formally and informally circumscribed its actions around what I refer to as the “Official Protocol,” consisting of targeted rather than universal lead screenings, a 20 µg/dL child blood lead level medical intervention threshold, soil identified as the primary pathway of exposure (thereby relegating paint, water, and air sampling), and intervention efforts focused squarely on what it considered the most urgent toxic “hot spots” located within squatter settlements housing the urban poor. Lead poisoning, briefly considered a universal scourge, was turned into a “ghetto disease” [9] reflected and reinforced through health officials’ oft-mentioned linkage of the “two p’s” of *plomo* (lead) and *pobreza* (poverty). The reasons for this shift in approach are complex, but I argue they were the result of political pressures that from the beginning sought to reduce the real and potential financial burden of enacting preventive care and tackling the root causes of lead contamination [6,7]. The outcomes of this shift to a pragmatic-based policy, however, are all too clear. It meant that no large-scale epidemiological study would ever be conducted in Uruguay, clinics and hospitals routinely denied blood lead exams to non-poor or non-squatter settlement residents, paints were rarely tested for lead because as one official told me, “The poor don’t paint their shacks”, tens of thousands of lead water pipe fixtures and connections remained in the country’s old houses and apartment buildings, and victims were widely blamed for their own poisoning [6,7].

In engaging with this research in Uruguay, I was struck by the parallel experiences of lead contamination in the global North and South. In countries ranging from the United States [9,10,11] to Argentina [12], Australia [13], China [14], El Salvador [15], France [16], Mexico [17], and Peru [18], to name only a few examples, one observes a similar pattern. Structural socio-economic conditions lead to the heightened exposure and differential risk of harm suffered by specialized workers and minority and poor children. Exposures may come from (among other sources) mining, smelters, the informal economy, e-waste recycling, paints, water, or TEL gasoline. Industries deny responsibility for, or the severity of, lead exposures and undermine scientific and public health definitions of thresholds of harm and intervention. The lead industries, along with willing political representatives and an at-times complicit media, circulate a public discourse that scrutinizes the poisoned children themselves by questioning the habits and values instilled in them by uneducated or negligent parents (poor hygiene, uncleanliness), ignorant and backward folk traditions (use of lead-glazed pottery or folk health remedies), the children’s own potentially pathological behaviors (eating paint chips or dirt by exhibiting pica), or the deviant and criminalized activities of parents working in the informal economy. Responsibility for contamination, then, shifts from industrial pollution, inadequate or negligent state regulation, or the broader structural forces of discrimination and inequality, to folk theories of the supposed cultural traits and deviant moral character of now pathologized individuals and subcultures. It is from within this broader social and political context that public health officials debate two potential responses to recognized lead hazards: to engage in primary prevention, universal screening and the elimination of lead sources; or to follow a path of harm reduction that includes targeted lead screenings, dust control, parental and child behavior modification and remediating toxic “hot spots.” The primary prevention response is initially more financially costly and elicits a powerful political and industrial backlash. The harm reduction approach is then portrayed as the only viable option; a more financially feasible and politically expedient “pragmatic” response. The public debate thus shifts from elimination of lead to definitions of “acceptable levels” of exposure, framed in reference to marginalized or deviant sub-populations who do not generate a lot of empathy [10].

As researchers have long recognized, the greatest public health strides in the struggle against lead poisoning have been made when the disease was considered a universal risk rather than one affecting particular sub-populations [9,10,11]. The dangers of pragmatism, or more specifically what Needleman [9] has termed an “enfeebled pseudopragmatism,” in guiding public health policy are multiple. Pragmatism tends to undermine the urgency of lead poisoning as a risk to all of society, through for instance reduced IQ and increased violence and aggression at the population-wide level. It pathologizes suffering by reducing it to certain marginalized sub-populations, fostering misguided “cultural” explanations that facilitate victim-blaming. It encourages the individualization of illness, drawing attention away from the structural determinants of health, including inequality-generating economic forces and institutionalized racism. Pragmatism, in short, often obscures rather than illuminates, while truncating meaningful action.

Lead contamination and poisoning will continue to be discovered around the world to the extent it is looked for and to the extent to which the conditions of discovery allow. These conditions typically include the presence of objective exposures combined with the status of local medical knowledge, the relative strength of activist or advocacy groups, a sympathetic media willing to narrate the lead story, and other social and political contexts that facilitate the recognition of environmental disease and a willingness to care for the afflicted [7,11]. Lead has been around long enough and continues to be economically useful enough to constitute both a legacy and an emergent toxicant in most parts of the world, although the degrees of each differ according to specific contexts. It may be more common to find acute cases of lead poisoning in the global South due to deficits in regulation or local scientific expertise and health care resources. These more acute cases may also arise due to shifting political economic forces under globalization, including the progressive transfer of hazardous industries and toxic trades from the global North to the South [19,20]. Transnational outsourcing and the transfer of toxic trades are themselves justified through pragmatism, with proponents arguing they bring desperately-needed jobs or income to economically depressed countries, or by the justification that environmental and health protections are “first world luxuries.” The opportunity in the face of continuing lead exposures and discoveries is that, unlike other chemical exposures that have been the subject of little toxicological or public health research, lead poisoning is the world’s most studied environmental disease, and scientific knowledge of its effects continuously develops. The challenge, as always, is to avoid the mistakes of the past, and to find ways to transcend the “enfeebled pseudopragmatism” that so often (mis)guides public health policy, both in the North and in the South. As long as the dangers of lead around the world persist, and they do not show encouraging signs of abetting, the dangers of pragmatism will lurk in their shadow. And as many have pointed out before, lead poisoning will tragically continue as a “grand experiment” where societies submit children as “unwitting subjects” of lead exposure, becoming the infamous “canaries in the mine” [10]. We can, and must, do better.
